# A peculiar lens-shaped structure observed in the South China Sea

**DOI:** 10.1038/s41598-017-00593-y

**Published:** 2017-03-28

**Authors:** Hongyang Lin, Jianyu Hu, Zhiyu Liu, Igor M. Belkin, Zhenyu Sun, Jia Zhu

**Affiliations:** 1 State Key Laboratory of Marine Environmental Science, and Department of Physical Oceanography, College of Ocean and Earth Sciences, Xiamen University, Xiamen, 361102 China; 2 Laboratory for Regional Oceanography and Numerical Modeling, Qingdao National Laboratory for Marine Science and Technology, Qingdao, 266237 China; 3 Graduate School of Oceanography, University of Rhode Island, Narragansett, RI, 02882 USA

## Abstract

Lens-shaped structures within thermocline potentially play a significant role in subsurface transport of mass, heat, and salt in the global ocean. Whilst such structures have been documented in many oceanic regions, none has been observed in the China Seas. This study reports on observations of a lens-shaped structure within thermocline in the southwestern South China Sea in September 2007. This structure had a maximum thickness of approximately 60 m and a horizontal extent exceeding 220 km. This lens was peculiar in that its size is larger than most similar structures documented in the literature. The lens core was characterized by well-mixed water with higher temperature (~28.8 °C), lower salinity (~33.3) and lower potential vorticity (PV) compared to the surrounding waters. Based on an ocean reanalysis, possible generation mechanism of the lens is explored by examining the evolution of surface and subsurface thermohaline properties, and an analysis of vertical PV flux. The lens was likely generated by a mixture of the local mixed-layer water and the water from the coastal jet separation site.

## Introduction

Subsurface lens-shaped structures have been known in the world’s oceans for decades. In particular, subsurface eddies characterized by a double-convex lens of well-mixed water were first documented in the 1970s–1980s and termed intrathermocline eddies (ITEs)^1–3^ or submesoscale vortices^4^. They have a velocity maximum in the interior and are often accompanied by compensated vertical shear of horizontal velocity above and below the lens^5^. Consequently sea surface manifestations of ITEs are normally much weaker than those of surface-intensified cyclones and anticyclones. Distinct characteristics of ITEs include weak stratification, anticyclonic circulation, a subsurface velocity maximum, and low potential vorticity (PV) within the core^6^. Water properties within ITEs are different from the surrounding waters, suggestive of remote origin of these ITEs. Therefore, they may play a significant role in the subsurface transport of mass, heat, and salt in the global ocean, and potentially impact global climate through carbon sequestration transporting carbon downward to the deep ocean.

Several generation mechanisms of lens-shaped structures have been proposed in the literature (see ref. 6 for a brief summary). For example, Spall^7^ demonstrated that they could be formed at upper-ocean fronts by baroclinic instability and frontogenesis, which subducts low-PV water on the cold side of the front along the frontal outcrop. Thomas^6^ showed, using numerical experiments, that frictional forces exerted by down-front winds can reduce PV within the frontal outcrop and provide a favorable condition for the formation of lens-shaped structures. Recently, McGillicuddy^8^ noted that the mesoscale lens in the thermocline could also be locally generated through eddy-wind interaction.

Lens-shaped structures have been observed in the thermocline in various regions of the ocean^5, 9–12^. Within the China Seas, Zhang *et al*.^13^ reported two subsurface lens-shaped water bodies in the South China Sea (SCS), and Xie *et al*.^14^ found a subsurface anticyclonic eddy in the Luzon Strait. However, these eddies were situated in the intermediate layer beneath the thermocline. Lens-shaped eddies have also been documented in the SCS based on numerical simulations and are considered to be caused by current-shelf interactions^15^. Yet, lens-shaped structures within the thermocline have rarely, if ever, been observed in the China Seas. In this study, we report on a peculiar lens-shaped structure in the SCS thermocline observed from *in situ* hydrographic measurements in September 2007. We will describe the characteristics of this lens-shaped structure, and shed some light on its possible generation mechanism.

The summertime upper-layer circulation in the SCS is driven primarily by the southwesterly monsoon wind and features a weaker cyclonic gyre north of about 12°N and a stronger anticyclonic gyre to the south^16, 17^. In between, an eastward jet separates from the coast of Vietnam^18^, and an eddy pair associated with the jet is formed. The generation mechanism of this eastward jet is still debatable; it has been attributed to the wind stress curl^18, 19^, coastal current-topography interactions^20^, or the confluence of two along-shelf flows near 11°–12°N^21^.

The coastal jet and eddy-pair structure are evident in the altimetry map of the absolute dynamic topography and associated surface geostrophic currents for the period of August 16–September 15, 2007 (Supplementary Fig. S1). Relevant to this offshore jet, a patch of cold surface water is found to separate from the nearshore region and veer northeastward to the deep sea. The monthly mean (August 16–September 15, 2007) wind field reveals a typical southwesterly summer monsoon with a wind stress of approximately 0.1 N m^−2^ upstream of the jet. The intensity of the coastal jet and the associated eddy pair is stronger in the map for September 7, 2007 (when the lens-shaped structure was observed), with a sea level difference across the jet reaching approximately 0.5 m (Supplementary Fig. S1). The coastal cold water is still visible in the daily sea surface temperature (SST) map, although it is confined to the nearshore area as an elongated filament. While the monthly wind field exhibits the dominating southwesterly monsoon, a divergent pattern is observed in the daily wind fields. Stronger alongshore winds dominate the nearshore region, whereas weaker westerly winds blow in the eastern offshore area with a magnitude less than 0.05 N m^−2^. The coastal cold jet corresponds well in location with the positive wind stress curl patch, suggesting that the cold SST might be due to upwelling induced jointly by the southwesterly monsoon (via offshore Ekman transport) and by positive wind stress curl (via upward Ekman pumping).

## Results

### Observation of a peculiar lens-shaped structure

**Figure 1:**
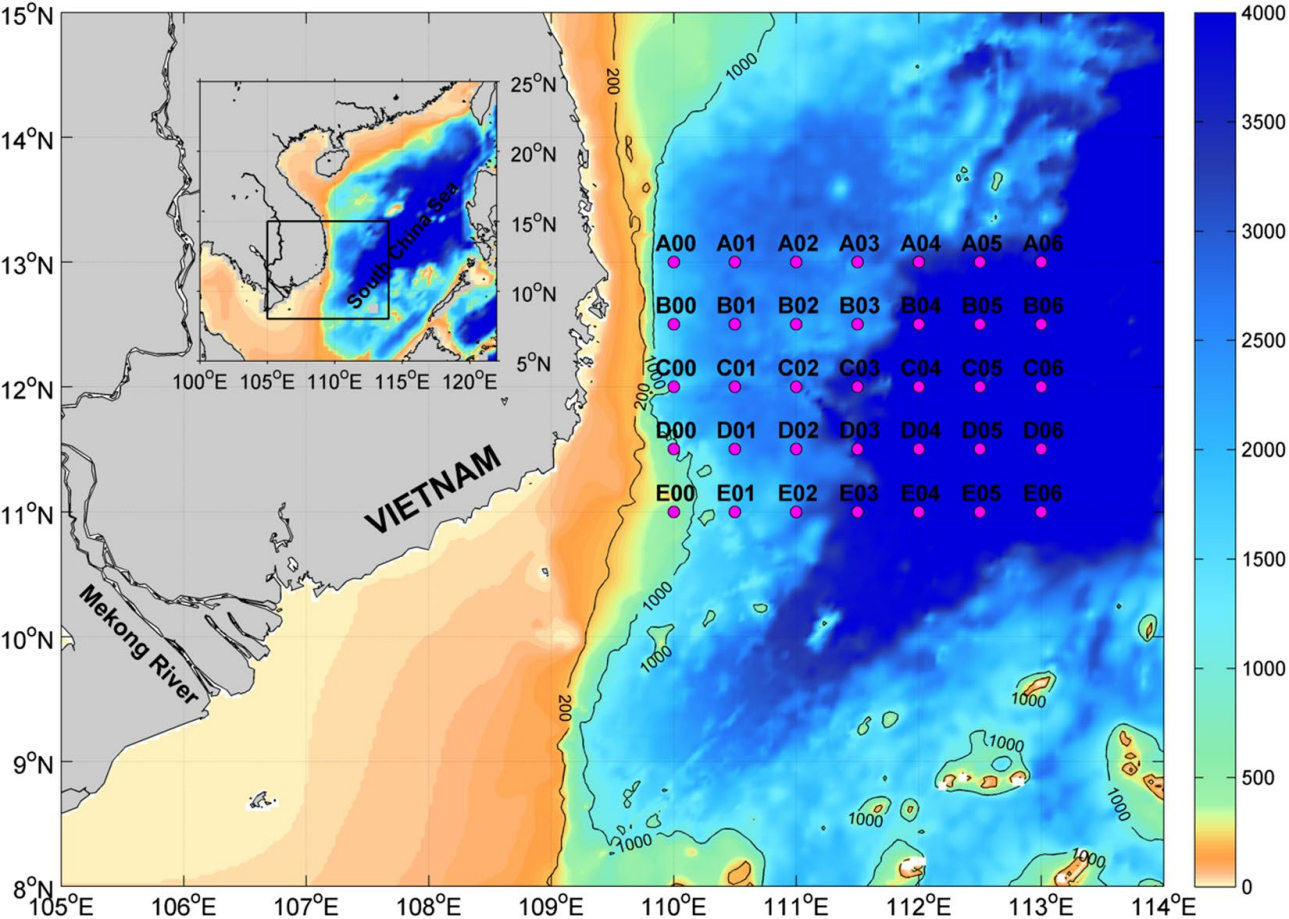
Map of the southwestern South China Sea. Bathymetry (ETOPO2, in m) is color coded superimposed with hydrographic stations (CTD casts, magenta dots). Inset shows the South China Sea with the zoom-in region shown by a box. The figure is generated using Matlab R2011a (www.mathworks.com/) with m_map package (www.eoas.ubc.ca/~rich/#M_Map).

Cruise measurements were conducted off the coast of southeastern Vietnam on September 2–8, 2007 onboard *R/V Dongfanghong II*. The sampling stations cover an area of 110°–113°E, 11°–13°N with a spatial interval of 0.5° in both longitude and latitude (magenta dots in Fig. 1). Detailed information about the *in situ* measurements is provided in Methods section.

**Figure 2:**
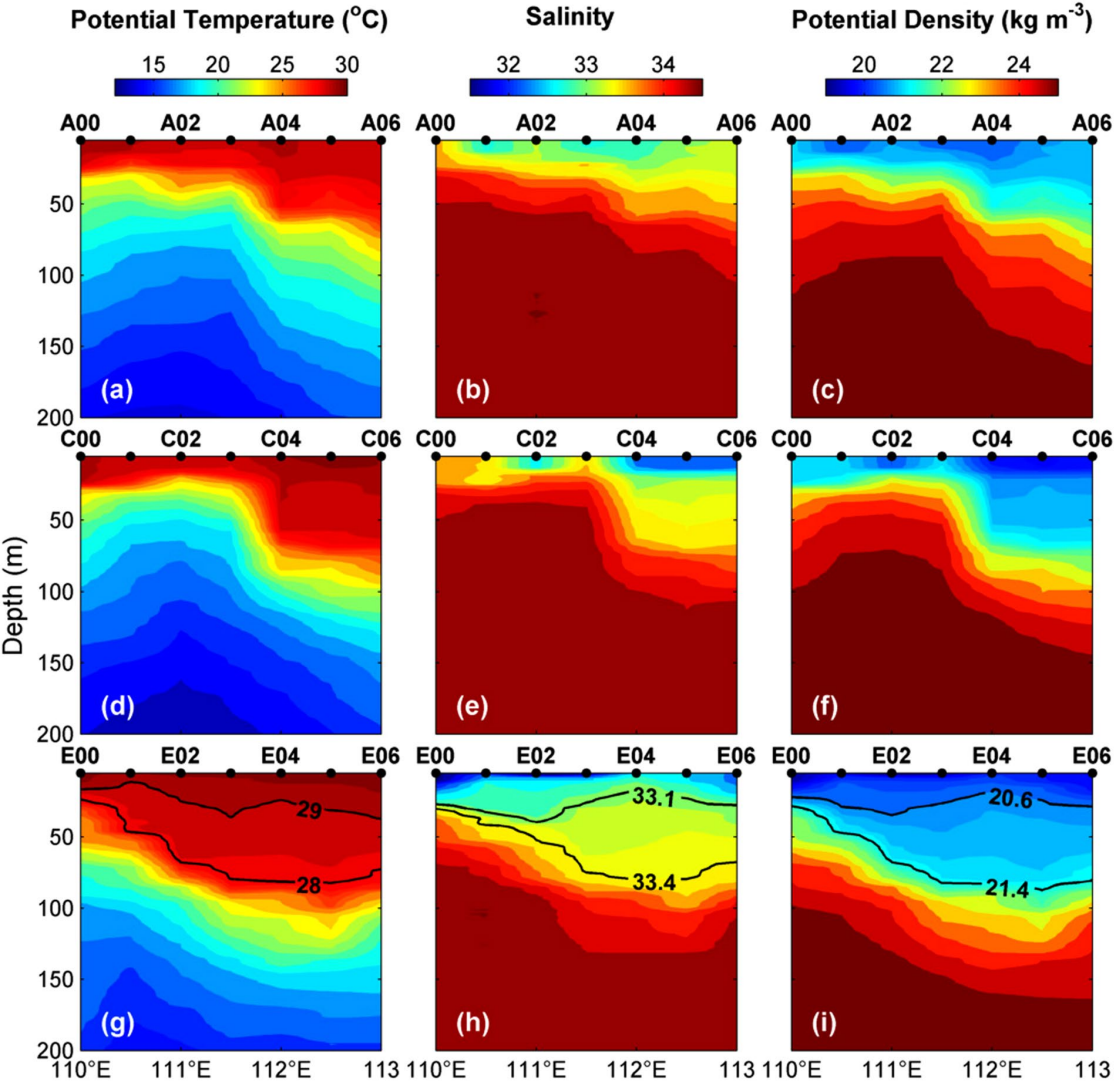
Sectional distributions of thermohaline properties. Potential temperature, salinity and potential density are shown in the left, middle and right columns of panels, respectively. The top, middle and bottom rows of panels are for transects A, C and E, respectively. The figure is generated using Matlab R2011a (www.mathworks.com/).

Vertical distributions of potential temperature (*θ*), salinity (*S*), and potential density (*σ*_*θ*_) are shown for three zonal transects (i.e., A00–A06, C00–C06 and E00–E06). Particular attention is paid to the southernmost transect where a peculiar lens-shaped structure was observed. Along transect A, the thermocline, halocline and pycnocline are tilted downward from west to east (Fig. 2a–c). Such a downward tilt toward east is in agreement with the sea level distribution (Supplementary Fig. S1d), which shows transect A extending from the periphery of a cyclonic eddy to an anticyclonic eddy. Although generally similar patterns are seen in transect C (Fig. 2d–f) as in transect A, e.g., shallower thermocline in the west and deeper in the east, there are also differences between the two transects, e.g., the slope of thermocline. The western half of transect C cuts across the center of cyclonic eddy; hence, a larger uplift of thermocline is observed compared to that at transect A. This also leads to a steeper pycnocline near station C03 (Fig. 2f), implying a larger horizontal density gradient and hence a stronger vertical shear of the velocity according to the thermal wind relation.

Only one (single-layer) thermocline/halocline/pycnocline is observed along transect A or C. In contrast, a double (two-layer) halocline/pycnocline is observed along transect E (Fig. 2g–i). The lower halocline (pycnocline), located at approximately 50–120 m, has a larger vertical gradient of *S* (*σ*_*θ*_) compared to the upper one. A lens of relatively well-mixed water is squeezed in-between the two high-gradient layers (“clines”) that define the upper and lower bounds of the lens-shaped structure. Notably, the lens shape is better defined in the vertical distributions of *S* and *σ*_*θ*_ than that of *θ*. To our best knowledge, lens-shaped structures within thermocline/pycnocline have not been documented in the SCS. The upper bound of the lens could be defined by the convex isohaline of 33.1 or by the isopycnal *σ*_*θ*_ = 20.6 kg m^−3^, and the lower bound is defined by the concave 33.4 isohaline or *σ*_*θ*_ = 21.4 kg m^−3^, so the largest thickness is about 60 m (from 20 to 80 m) near station E04 (Fig. 2h). There is evidence that transect E does not fully cut across the entire lens-shaped structure, which is expected to extend further east than E06 (Fig. 2h and i). In other words, the horizontal extent of the lens is larger than 220 km (the distance from E02 to E06). This is another peculiar feature of the observed lens-shaped structure, i.e., its horizontal size is larger than most subsurface lenses documented in the literature. We suspect that the anomalous size is due to the larger Rossby radius of deformation in the study region, and more discussion is provided in the final section.

Vertical profiles of thermohaline properties indicate that above the main thermocline (~80 m), the lens-shaped structure has clearly the highest *θ*, lowest *S* and hence lowest *σ*_*θ*_ amongst all profiles (Supplementary Fig. S2). Specifically, the lens-shaped structure is characterized by a water mass with a thermostad of ~28.8 °C, a halostad of ~33.3 and a pycnostad of ~20.9 *σ*_*θ*_ between 20 and 80 m. The lens-shaped structure corresponds to the dot cluster in the upper right corner of the *θ*–*S* diagram, exhibiting a relatively isolated feature compared to the ambient waters.

**Figure 3:**
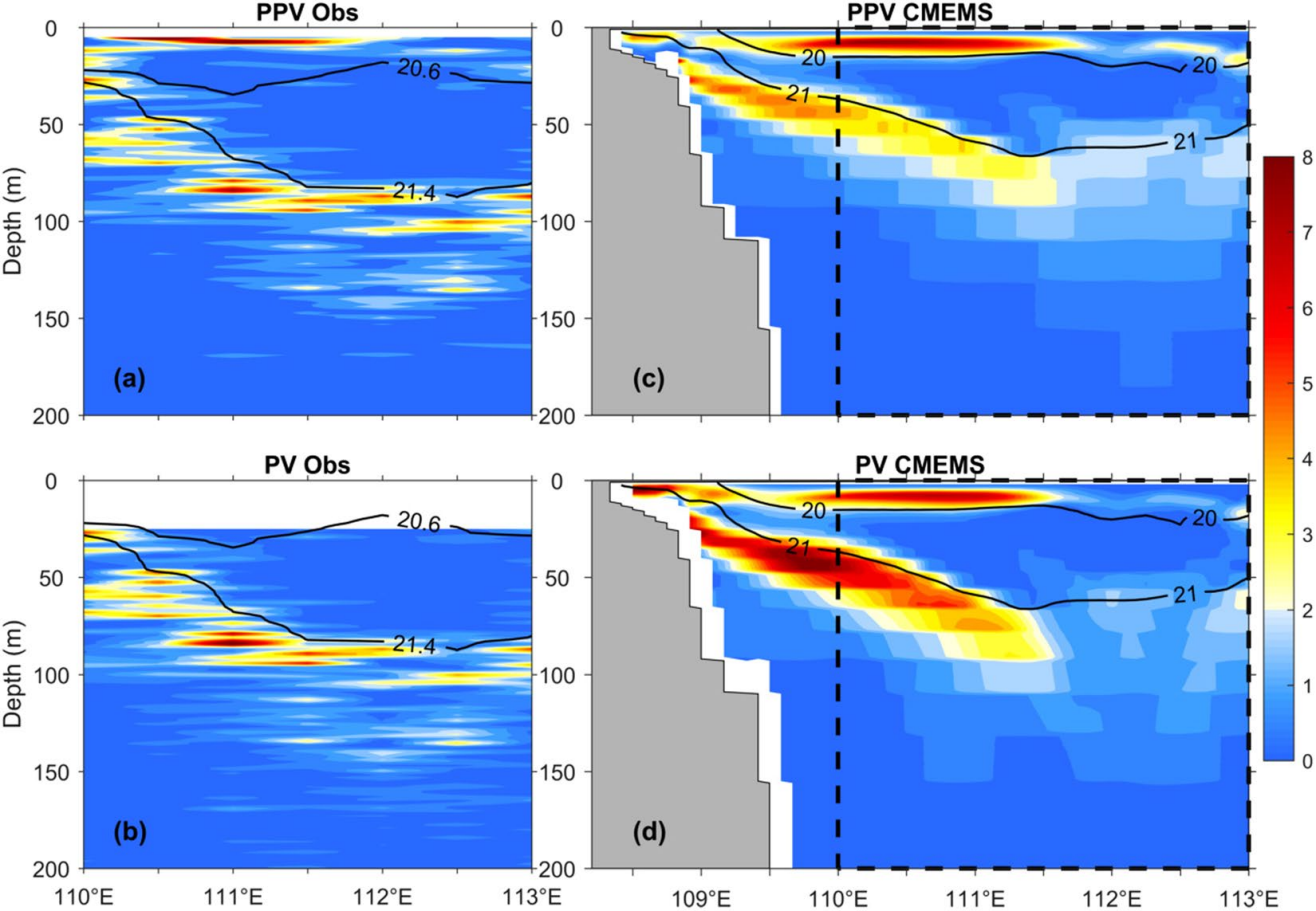
Potential vorticity (PV; ×10^−9^ m^−1^ s^−1^) along transect E based on observations and CMEMS reanalysis. Top: Planetary PV (PPV). Bottom: absolute PV. PV values are color coded. Isopycnals (black contours) bound the lens-shaped structure (20.6 *σ*_*θ*_ and 21.4 *σ*_*θ*_ for observations as Fig. 2i; 20 *σ*_*θ*_ and 21 *σ*_*θ*_ for reanalysis as Fig. 4i). The *in situ* velocities are only available below 24.7 m, and hence the blank area is shown above ~25 m in (**b**). The dashed boxes (right column) represent the area where observed data were collected. The figure is generated using Matlab R2011a (www.mathworks.com/).

The PV is also calculated along the vertical section of transect E, which is defined by:

1 $$PV=-\frac{(f+\zeta )}{\rho }\frac{\partial \rho }{\partial z}\,$$

where $\zeta =\frac{\partial v}{\partial x}-\frac{\partial u}{\partial y}$ is the vertical component of the relative vorticity, *f* is the Coriolis parameter, and *ρ* is the potential density. PV reduces to planetary PV (PPV) when *ζ* is negligible^22^. It is clear that PV is largely dominated by PPV (Fig. 3a,b) and a lens of low PV also exists in the area where the observed lens-shaped structure is situated (Fig. 3a). Large PV values are found near sea surface and in the vicinity of the main thermocline. This lens of low PV results primarily from the weak stratification, in agreement with results shown in Fig. 2.

### Validation of CMEMS reanalysis

In order to investigate the dynamics of the observed lens-shaped structure, we employ an ocean reanalysis product (hereinafter CMEMS reanalysis; see Methods for more information about the reanalysis). As will be shown below, CMEMS reanalysis is able to reproduce many observed features in the study region, both horizontally and vertically.

The northeastward jet off the Vietnam coast observed from altimetry is well reproduced by CMEMS reanalysis in both monthly averaged and daily maps (Supplementary Fig. S1). The eddy pair associated with this jet also resembles the observed one in the monthly averaged field. More surprisingly, the anticyclonic eddy located to the northeast of the eddy pair is also seen in the reanalysis. The SST fields exhibit larger differences with the observations compared to sea level fields, but the large-scale patterns are qualitatively similar (e.g., the coastal cold water patch extending northeastward).

**Figure 4:**
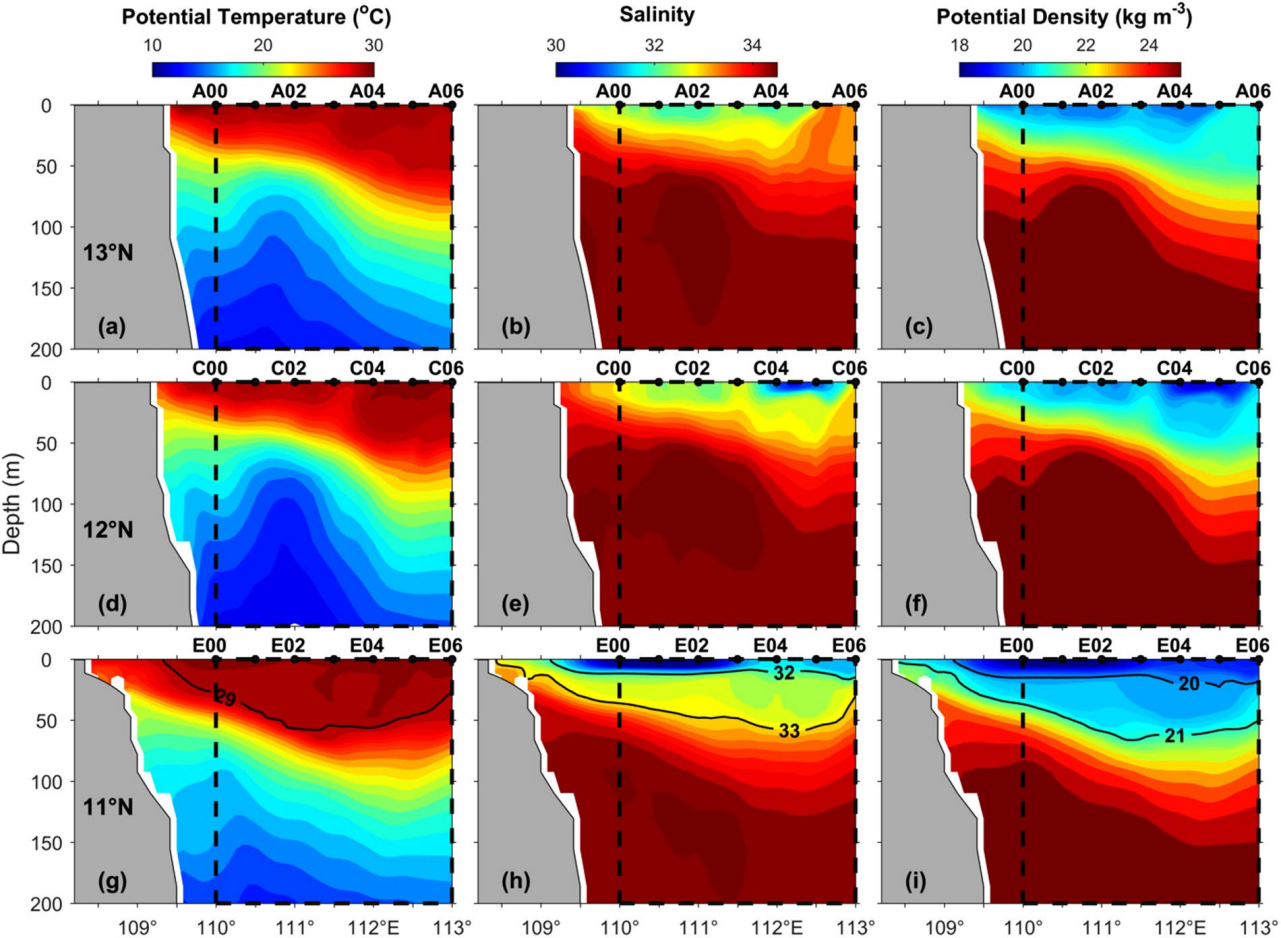
Sectional distributions of thermohaline properties based on CMEMS reanalysis. Similar format as Fig. 2 except that the observations are only confined to the east of 110°E as bounded by the dash box in each panel of this figure. The figure is generated using Matlab R2011a (www.mathworks.com/).

There is fair agreement in sectional distributions of *θ*, *S* and *σ*_*θ*_ along the above three zonal transects (A, C and E) between the observations and CMEMS reanalysis (Fig. 4), suggesting its good performance on vertical structures. For example, both the observations and reanalysis exhibit the downward tilt of thermocline/halocline/pycnocline (“clines”) toward east along transect A (13°N), and the steeper “clines” along transect C (12°N). More importantly, a lens-shaped structure enclosing a relatively homogenous water mass is also seen in the reanalysis data along transect E (11°N) as observed in Fig. 2. Just like the observations, the lens-shaped structure is better defined in the vertical distributions of *S* and *σ*_*θ*_ than that of *θ*, although the upper and lower bounds of the lens are characterized by different isohalines (32 and 33) and isopycnals (*σ*_*θ*_ = 20 kg m^−3^ and 21 kg m^−3^) compared to the observations. Given the similar thermohaline structures (Figs 2 and 4), it is expected to see a lens of relatively low-PV water along transect E in the reanalysis data (Fig. 3c,d), which is qualitatively similar to the observations (Fig. 3a,b).

According to the comprehensive comparisons, it is plausible to infer that the CMEMS reanalysis is able to fairly well reproduce many observed features in the study region including the lens-shaped structure. This gives us confidence to further investigate the dynamics of the observed lens-shaped structure using this reanalysis. One advantage of using the reanalysis product is that it provides continuous high-resolution data that allow us to examine the evolution of particular features. Another advantage is that the reanalysis fills the gap where the *in situ* measurements are not available. For example, vertical structures of thermohaline properties in the nearshore region (west of 110°E) are crucial in understanding the origin and generation of the observed lens-shaped structure, but this area was not covered by observations which could be overcome with the aid of the reanalysis.

### Generation mechanism

**Figure 5:**
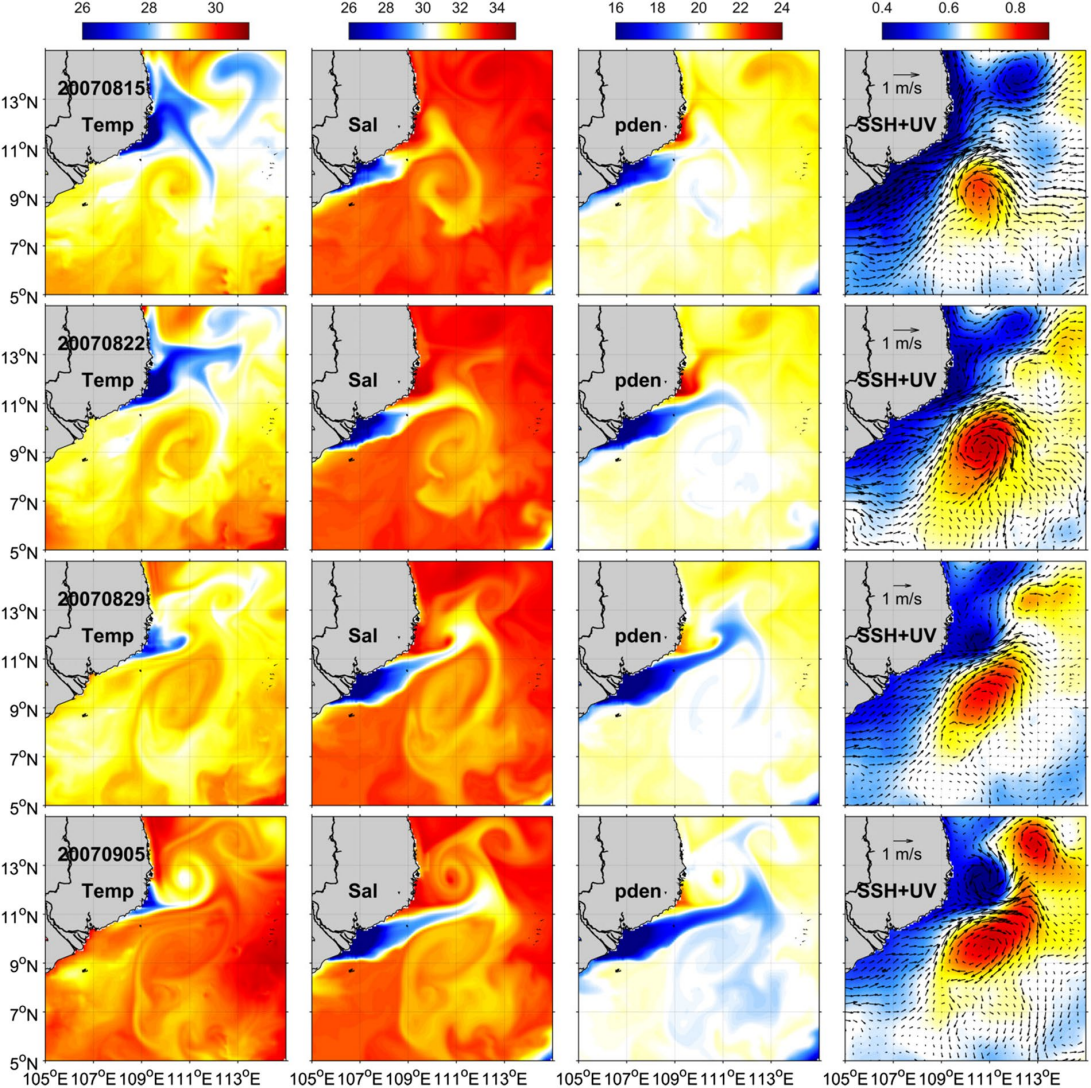
Weekly snapshots of the sea surface thermohaline and circulation patterns. Left to right: sea surface temperature (ºC), salinity, potential density σ_θ_ (kg m^−3^) and sea surface height (color; with current vectors superimposed). Each row is for a particular date which is labelled in the leftmost panel. The figure is generated using Matlab R2011a (www.mathworks.com/) with m_map package (www.eoas.ubc.ca/~rich/#M_Map).

Possible generation mechanism of the lens-shaped structure is now investigated based on CMEMS reanalysis. The sectional distributions indicate that the lens-shaped structure is capped by a patch of low-salinity water (Figs 2h and 4h), which is probably from the Mekong River. We thus examine the evolution of the surface thermohaline and circulation patterns (Fig. 5).

On August 15, 2007 (three weeks before the lens-shaped structure was observed) the fresh water was confined to the river mouth and a large patch of cold water occupied the coast of Vietnam north of 11°N. There was only one anticyclonic eddy located south of the coastal jet (Fig. 5). The vertical section along 11°N at this time suggested a well-mixed layer above 70 m and no lens-shaped structure was seen (Supplementary Fig. S3). In the next two weeks the river plume extended northeastward, forming an elongated filament, whereas the coastal cold water retreated to the very nearshore area. A cyclonic eddy was gradually generated to the north of the jet, forming the eddy pair (Fig. 5). The vertical section along 11°N suggested that a thin fresh layer occupied the sea surface to the west of ~111°E, and the original mixed-layer water seemed to be situated underneath and be capped by the surface fresh water (Supplementary Fig. S3). On September 5, the elongated fresh filament took a straight path and extended to ~113°E, and the eddy pair became the strongest (Fig. 5). The lens-shaped structure was also clearly seen in the vertical section along 11°N (Supplementary Fig. S3). The evolution of the surface patterns and sectional distributions implies that the lens-shaped structure possibly stems from the mixed-layer water, which was capped by a thin fresh layer originated from the Mekong River.

Sectional distributions of salinity and potential density along 11°N also reveal that the isohalines and isopycnals bounding the lens-shaped structure tilt upward toward west and outcrop in the nearshore area (Fig. 4h,i), which was missed by observations. This prompts us to speculate that the low-PV water within the lens (Fig. 3) could also originate from the outcropped region, i.e., the surface low-PV water subducts along tilted isopycnals. This is further examined based on a diagnostic analysis of the vertical PV flux. Normally the formation of low-PV water depends crucially on two PV flux components, ${J}_{z}^{D}$ and ${J}_{z}^{F}$, associated with diabatic processes and external frictional forces (i.e., wind stress), respectively (see Methods for derivation of ${J}_{z}^{D}$ and ${J}_{z}^{F}$).

The vertical PV flux associated with diabatic processes (${J}_{z}^{D}$) exhibits a distinct negative extremum outside the Mekong River estuary (Supplementary Fig. S4). This is related to the large fresh water flux into the ocean, increasing the local stratification and resulting in PV gains. Over most of the southern SCS, the magnitudes of ${J}_{z}^{D}$ are negligible including the location of the observed lens. In particular, values of $\,{J}_{z}^{D}$ are primarily negative if not close to zero, so the distribution of ${J}_{z}^{D}$ is not favorable to the formation of the observed low-PV water. The vertical PV flux associated with frictional forces (${J}_{z}^{F}$) reveals a negative band extending northeastward from the Mekong estuary to the western part of transect E. In contrast, a patch of positive ${J}_{z}^{F}$ is found in the nearshore region to the west of transect E (Supplementary Fig. S4). The maximum value occurs near the separation site of the coastal jet (near 11°N, 109°E; Supplementary Fig. S1), suggesting a strong wind-induced upward PV flux (and hence a PV reduction) at this site. Moreover, ${J}_{z}^{F}$ is nearly one order of magnitude larger than ${J}_{z}^{D}$ in the area where ${J}_{z}^{F}$ is positive. In short, the coastal jet separation site, which is also the outcropped window of the observed lens-shaped structure, undergoes significant wind-induced PV losses, and might thus serve as a low-PV water source for the observed lens through isopycnal subduction. This is in fact the “down-front” wind mechanism^6^, i.e., the southwesterly monsoon (Supplementary Fig. S1) blows on top of the northeastward coastal jet, leading to the destruction of PV locally.

**Figure 6:**
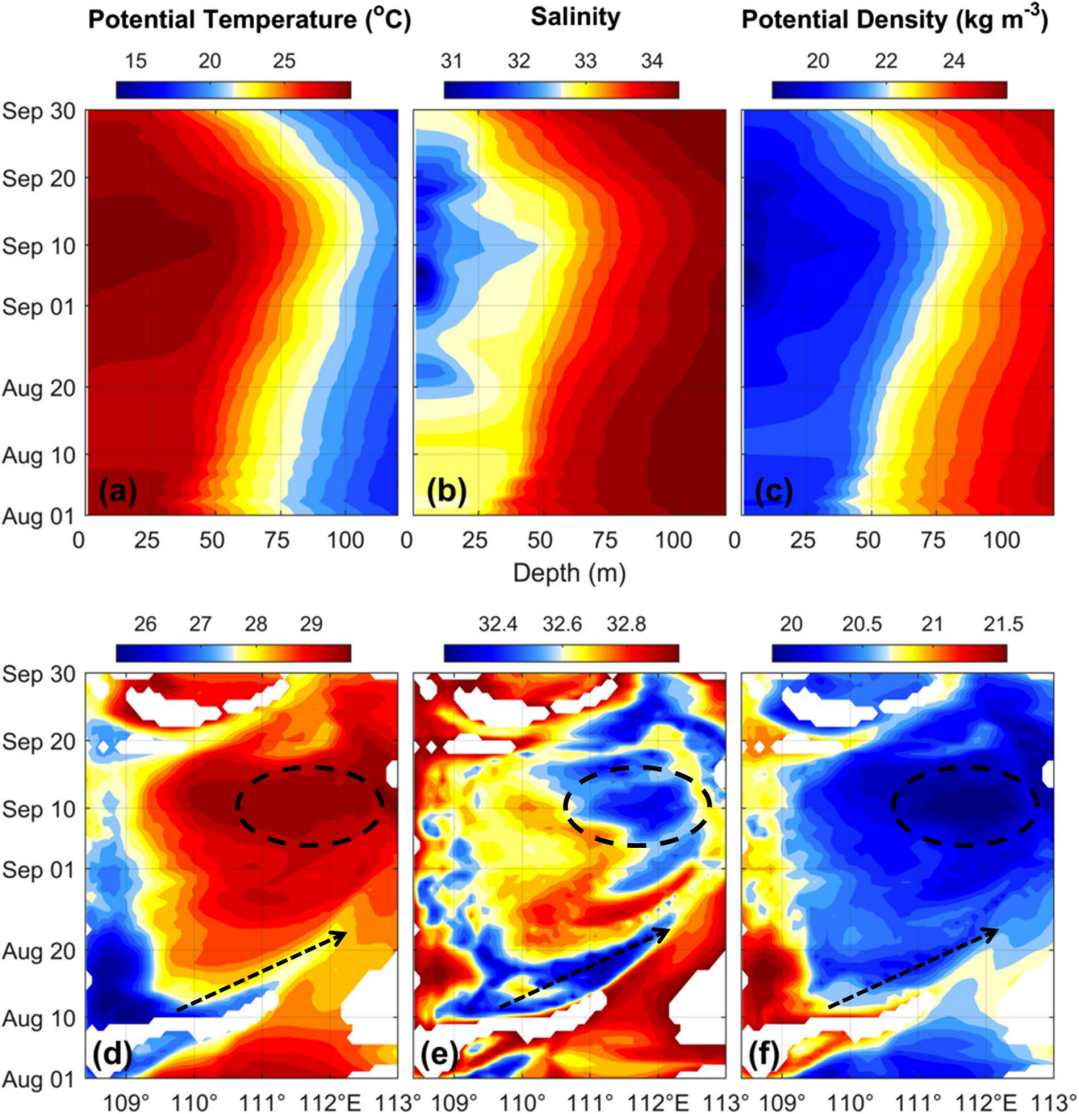
Hovmöller plots of thermohaline properties. Top: time–depth plots of (**a**) *θ*, (**b**) *S* and (**c**) *σ*_*θ*_ horizontally averaged between 111°–113°E. Bottom: time–longitude plots of (**d**) *θ*, (**e**) *S* and (**f**) *σ*_*θ*_ vertically averaged between isohalines 32 and 33. Dashed arrows denote eastward propagation; dashed circles indicate the stagnant waters. Blank indicates sea surface salinity higher than 33. The figure is generated using Matlab R2011a (www.mathworks.com/).

The above analysis suggests two origins for the observed lens-shaped structure: i) local mixed layer, and ii) coastal jet separation site (or the outcropped window). To pinpoint the origin, we examine the Hovmöller diagrams of thermohaline properties for each case following previous studies^23, 24^. Since the lens-shaped structure is most prominent in the 111°–113°E range, we thus horizontally average *θ*, *S* and *σ*_*θ*_ at each depth and investigate their evolution with depth. There is a clear deepening of mixed-layer water from August 1 to September 12 and a shallowing retreat after that (Fig. 6a–c). From late August, a patch of fresh water started to occupy the sea surface and the lowest salinity appeared at the sea surface when the lens-shaped structure was observed at subsurface (Fig. 6b). The time-depth plots are consistent with the above results (Fig. 5, Supplementary Fig. S3) and support the hypothesis that the observed lens-shaped structure stemmed from the mixed-layer water prior to being capped by the Mekong River plume. Figure S3 indicates that the lens is relatively well bounded by isohalines of 32 and 33 over time. We thus vertically average *θ*, *S* and *σ*_*θ*_ between these two isohalines along 11°N and examine their evolution with longitude. There is evidence of eastward propagation from August 10 to early September (see dashed arrows in Fig. 6d–f). A patch of warm and fresh water seems to be stagnant in 111°–113°E from early to middle September (dashed circles in Fig. 6d–f), which is in fact the water within the lens-shaped structure. It is thus plausible to infer that the low-PV water in the vicinity of the outcropped window could become subducted along sloping isopycnals so as to feed the lens, but this process seems to be relatively short-lived and intermittent.

Therefore, the Hovmöller plots support the hypothesis that the lens-shaped structure might be generated by a mixture of two origins: i) the local mixed-layer water and ii) the water near the outcropped window.

## Discussion

A mesoscale lens-shaped structure was observed in the southwestern SCS from cruise measurements conducted off the coast of Vietnam in September 2007. The lens-shaped structure has a largest thickness of approximately 60 m and a horizontal extent exceeding 220 km. It encloses well-mixed waters with high temperature (~28.8 °C), low salinity (~33.3), low potential density (~20.9 *σ*_*θ*_) and also low potential vorticity (PV) compared to the surrounding waters.

With the aid of CMEMS reanalysis, we explore the generation mechanism of the observed lens-shaped structure based on the evolution of surface and sectional thermohaline properties, and an analysis of vertical PV flux. We propose that the observed lens is generated by a mixture of two origins. First, the water within the lens stems largely from the local mixed-layer water which was capped by the Mekong River plume. Second, the water near the separation site of coastal jet, which serves as an outcropped window, could also intermittently feed the lens by isopycnal subduction influenced by the “down-front” wind.

Further examination of the sectional distribution of thermohaline properties over a longer period and a smaller time interval (similar to Supplementary Fig. S3; figures not shown) suggests that the first mechanism mentioned above would be more likely. The nearshore outcropped window changes its area considerably with time and sometimes even disappears. The evolution of the sectional maps based on CMEMS reanalysis also indicates that after the retreat of Mekong River plume, the subsurface low-PV water was exposed to the sea surface again. So we believe the observed lens was more likely to have formed from the original mixed-layer water. More sophisticated observations and modelling studies are needed to verify this process.

The radius of the observed lens-shaped structure (>110 km) is larger than most similar structures (~tens of km) documented in previous studies. The local mixed-layer deformation radius is determined by *L = Nh/f* ^25^, with *N*, *h* and *f* denoting buoyancy frequency, mixed-layer depth and the Coriolis parameter, respectively. *N* is ~7.1 × 10^−3^ s^−1^ within the lens based on the conductivity-temperature-depth (CTD) measurements, and *h* is ~60 m, lens thickness. This gives a value of *L* of approximately 15 km, which is smaller than the observed size by a factor of 7~8, suggesting that the horizontal scale of the lens-shaped structure was not determined by the mixed-layer instabilities. In contrast, the first baroclinic Rossby deformation radius near the region where the lens was observed is ~80 km^26^. Suppose a water mass initially has a horizontal scale close to Rossby deformation radius, and this water mass becomes subducted to form the observed lens-shaped structure. If it is squashed during subduction due to PV conservation, its horizontal size is expected to expand as constrained by volume conservation, and then its anomalously large size could be explained. However, process-oriented numerical modeling studies are required to infer a more definitive explanation.

Although the observed subsurface lens-shaped structure seems to exist for a relatively short period (the lens existed until September 15, figures not shown), subduction of surface water into the ocean interior might still have a broader implication on the regional biogeochemistry. Observations have identified significant CO_2_ drawdown related to the Amazon River plume^27^, and higher nitrogen fixation associated with the Mekong River plume^28^. Will the subsurface lens-shaped structures play a role in this regard ? To answer this question, a comprehensive study is required. More *in situ* measurements are needed to verify the possible recurrence of the lens-shaped structure in the study area. Three-dimensional observations extending from the Mekong River estuary to the coastal jet separation site and to the observed lens location are required to identify the origin and dynamics in a more definitive way.

## Methods

### *In situ* data

At each sampling station (see magenta dots in Fig. 1), measurements of temperature, salinity and currents were made by a Seabird SBE 911 CTD profiler, and a ship-board acoustic Doppler current profiler (ADCP). After quality control, the CTD data were interpolated onto vertical grids with 1 m spacing. The underway ADCP data have a time interval of 3 min and a bin size (vertical spacing) of 16 m with the first bin located at 24.7 m below the sea surface. The measurements were conducted on September 2–8, 2007, and Transect E (i.e., the southernmost transect E00–E06; Fig. 1) was sampled from late September 7 to September 8, 2007.

### Satellite data

Satellite products used in the present study include sea level, SST, and wind fields. The sea level data were based on altimeter observations obtained from Archiving, Validation, and Interpretation of Satellite Oceanographic data (Aviso). The Aviso daily products of absolute dynamic topography and the associated geostrophic velocities, both with a grid spacing of 0.25°, were used in this study (http://www.aviso.altimetry.fr/). Satellite-based SST data were the Optimally Interpolated products (mw_ir) produced by Remote Sensing Systems (RSS; http://www.remss.com/) and distributed by the Group for High Resolution Sea Surface Temperature (GHRSST; http://data.nodc.noaa.gov/ghrsst/L4/GLOB/REMSS/mw_ir_OI/). This SST product was provided daily with a spatial resolution of approximately 9 km. Surface wind fields for the SCS were obtained from QuikSCAT scatterometer measurements provided by RSS (http://www.remss.com/missions/qscat/). Daily mean products were used with a spatial resolution of 0.25°.

### Reanalysis datasets

The reanalysis datasets used include the global ocean physics analysis and forecast system (GLOBAL_ANALYSIS_FORECAST_PHY_001_024) distributed by the Copernicus Marine Environment Monitoring Service (CMEMS; http://marine.copernicus.eu/). This dataset, termed CMEMS reanalysis in this study for brevity, is distributed daily and has a horizontal resolution of 1/12° in both longitude and latitude, 50 vertical levels ranging from 0 to 5700 m, including 27 levels in the upper 220 m. This reanalysis is based on NEMO (Nucleus for European Modelling of the Ocean) with sophisticated data assimilations. For more detailed information about the reanalysis the reader is referred to its user manual (http://marine.copernicus.eu/documents/PUM/CMEMS-GLO-PUM-001-024.pdf). The Simple Ocean Data Assimilation (SODA^29^) is also used in the present study, including monthly temperature, salinity, surface heat flux and net fresh water flux for August 2007 with a horizontal resolution of 0.5°.

### Estimation of PV flux

PV dynamics are briefly reviewed in order to derive the PV flux induced by diabatic and frictional forcing. A more detailed analysis of PV dynamics could be found in, for example, refs 6, 22 & 30. The flux form of mass-weighted PV equation can be expressed as:

2 $$\frac{\partial (\rho q)}{\partial t}=-\nabla \cdot {\bf{J}}\,$$

where

3 $$q=-\frac{1}{\rho }{{\boldsymbol{\omega }}}_{{\boldsymbol{a}}}\cdot \nabla {\sigma }_{\theta }\,$$

is the full Ertel PV and ${{\boldsymbol{\omega }}}_{{\boldsymbol{a}}}=f{\boldsymbol{k}}+\nabla \times {\boldsymbol{u}}$ is the absolute vorticity with ***k*** being the vertical unit vector and ***u*** the velocity. The PV flux **J** is determined by,

4 $${\bf{J}}=\rho q{\boldsymbol{u}}+{{\boldsymbol{\omega }}}_{{\boldsymbol{a}}}\frac{D{\sigma }_{\theta }}{Dt}+{\bf{F}}\times \nabla {\sigma }_{\theta }\,$$

where **F** represents frictional forces per unit mass. Substituting (4) into (2) we obtain,

5 $$\frac{\partial (\rho q)}{\partial t}+{\boldsymbol{u}}\cdot \nabla (\rho q)=-{{\boldsymbol{\omega }}}_{{\boldsymbol{a}}}\cdot \nabla (\frac{D{\sigma }_{\theta }}{Dt})-\nabla \times {\bf{F}}\cdot \nabla {\sigma }_{\theta }\,$$

The first term on the right hand side of equation (5) represents the PV flux arising from the diabatic processes associated with air-sea buoyancy flux. The second term represents the contribution from frictional forces assumed to arise primarily from external wind forcing.

The PV flux is illustrated by considering a control volume enclosing the lens-shaped structure bounded by two isopycnals outcropping at the sea surface, which serves as a window connecting the lens with the atmosphere. A schematic of such a control volume could be found in, for example, Fig. 2 of ref. 31, or Fig. 1 of ref. 6. According to the “impermeability theorem”^32^, there is no PV flux across the isopycnals, thus the only PV flux of the control volume would occur at the outcropped region. A positive (or outward) PV flux at the sea surface reduces the volume-integral PV and would possibly lead to the formation of the low-PV lens.

We thus consider the vertical component of the PV flux through the mixed layer associated with the ventilation process and ultimately the lens formation^22^. The vertical component of the PV flux averaged over the mixed layer is,

6 $${J}_{z}=\frac{1}{h}{\int }_{-h}^{0}{\bf{J}}\cdot {\boldsymbol{k}}dz\,$$

where *h* is the mixed-layer depth. After a set of manipulations detailed in ref. 22, the PV flux component associated with diabatic processes is derived as,

7 $${J}_{z}^{D}=-\frac{f}{h}(\frac{\alpha {Q}_{net}}{{c}_{w}}+{\rho }_{0}\beta S{F}_{net})$$

where *Q*_*net*_ and *F*_*net*_ are net air-sea heat flux and net fresh water flux, respectively, *α* and *β* are the thermal expansion and saline contraction coefficients, *c*_*w*_ is the isobaric heat capacity of sea water. By obtaining equation (7), we have assumed that the diabatic component is dominated by vertical mixing and thus could be evaluated by the divergence of buoyancy flux^6, 22^, represented by terms inside the bracket of equation (7) following Marshall and Schott^33^. The component associated with frictional forces is^22^,

8 $${J}_{z}^{F}=(\frac{{{\boldsymbol{\tau }}}^{{\boldsymbol{s}}}}{{\rho }_{0}h}\times \nabla {\sigma }_{\theta })\cdot {\boldsymbol{k}}\,$$

assuming the stress at the base of mixed layer is negligible and ***τ*** ^*s*^ is surface (wind) stress.

Calculation of ${J}_{z}^{D}\,\,$uses the monthly SODA data for August 2007 to estimate coefficients *α*, *β*, *Q*_*net*_, *F*_*net*_, and the mixed-layer depth *h* (based on the ${\rm{\Delta }}{\sigma }_{\theta }$ = 0.125 kg m^−3^ criterion^34^). Calculation of ${J}_{z}^{F}$ requires estimates of wind stress and surface density gradients. We estimate ∇*σ*_*θ*_ averaged over two weeks (from August 26 to September 8, 2007) before transect E was sampled using CMEMS and SODA reanalysis datasets. The wind stress, averaged over the same period, is computed based on QuikSCAT wind vectors^35^.

## Electronic supplementary material

Supplementary Information
