# Supplementary material for: A peculiar lens-shaped structure observed in the South China Sea
Source: Sci Rep. 2017 Mar 28;7:478. doi: 10.1038/s41598-017-00593-y (PMC5428710; doi:10.1038/s41598-017-00593-y)
Supplement: Supplementary file 1 — Supplementary Information [file 41598_2017_593_MOESM1_ESM.pdf]

Supplementary Information:

A peculiar lens-shaped structure observed in the South China Sea

Hongyang Lin[1], Jianyu Hu[1*], Zhiyu Liu[1,2], Igor M. Belkin[3], Zhenyu Sun[1] & Jia Zhu[1]

1. State Key Laboratory of Marine Environmental Science, and Department of Physical Oceanography, College of Ocean and Earth Sciences, Xiamen University, Xiamen 361102, China

2. Laboratory for Regional Oceanography and Numerical Modeling, Qingdao National Laboratory for Marine Science and Technology, Qingdao 266237, China

3. Graduate School of Oceanography, University of Rhode Island, Narragansett, RI 02882, USA

Corresponding author address: Dr. J. Hu, College of Ocean and Earth Sciences, Xiamen University, Xiamen, Fujian 361102, China.

Email: hujy@xmu.edu.cn

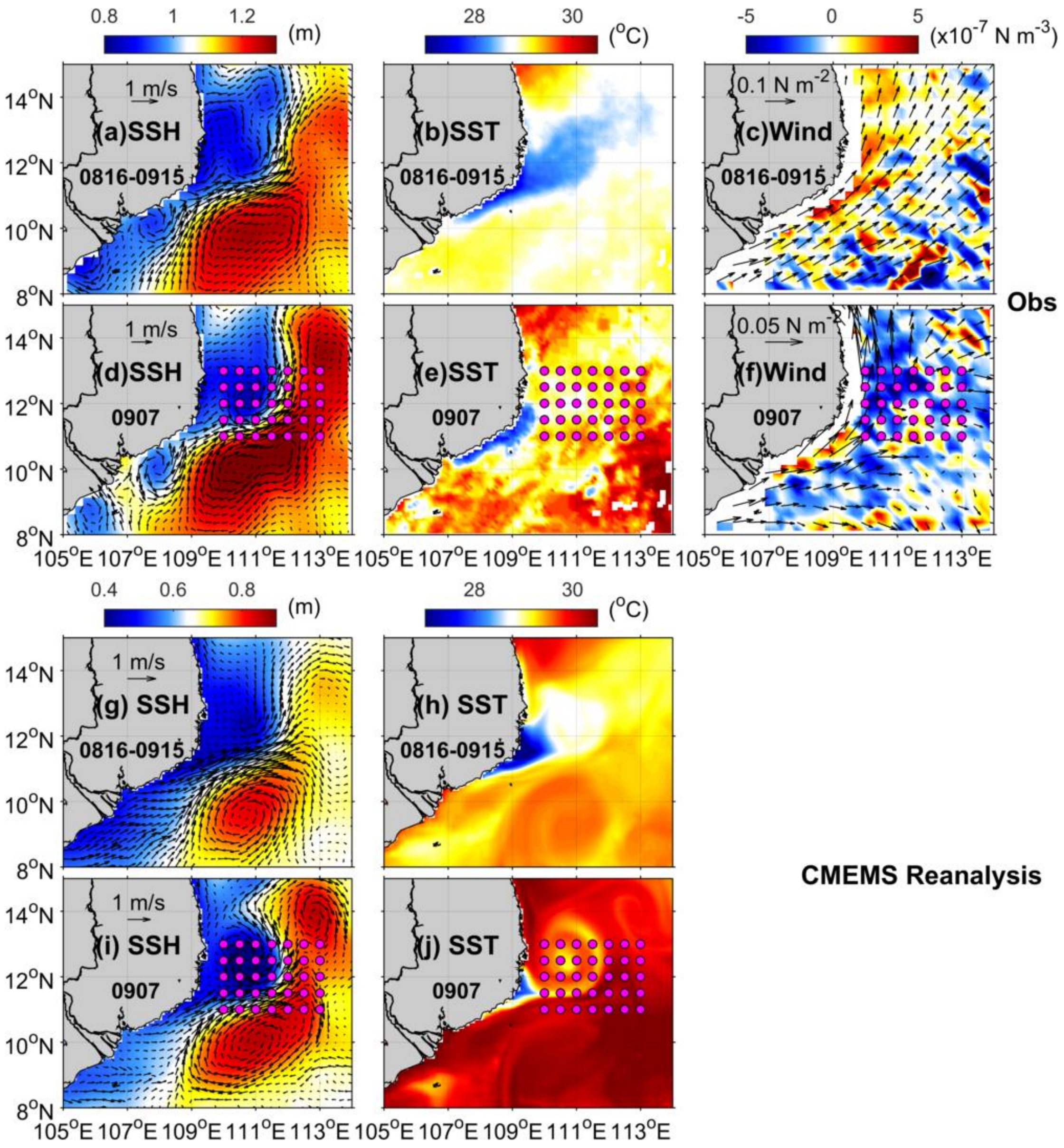

Figure S1. Maps of sea surface height (SSH), sea surface temperature (SST) and winds in the southwestern South China Sea. Upper two rows of panels show satellite observations and the lower two rows are based on CMEMS reanalysis. Monthly averaged (August 16 to September 15, 2007) and daily maps (September 7) are shown for both datasets. In the leftmost panels, vectors denote surface geostrophic currents; in panels c and f, vectors denote wind stress and color shading denotes wind stress curl. Hydrographic stations are indicated by magenta dots. The figure is generated using Matlab R2011a (www.mathworks.com/) with m_map package (www.eoas.ubc.ca/~rich/#M_Map).

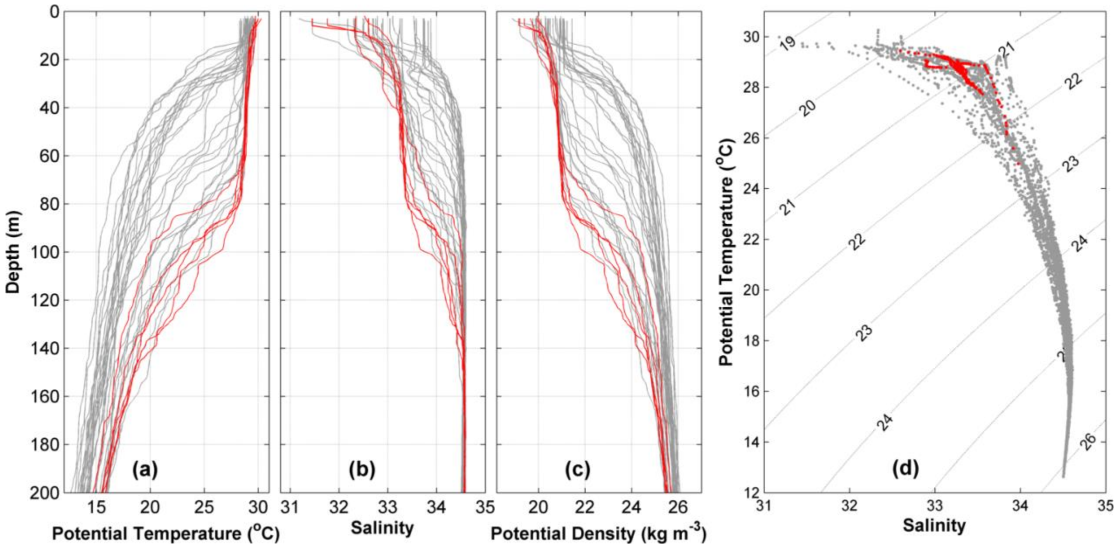

Figure S2. Vertical profiles of observed thermohaline properties. Vertical profiles of (a) potential temperature, (b) salinity, and (c) potential density based on *in situ* measurements from all sampling stations. Profiles obtained from stations E02–E06 are shown in red, otherwise in gray. (d) Potential temperature-salinity diagram based on the measurements. Data points within the lens-shaped structure (20–80 m in stations E02–E06) are shown in red, otherwise in gray. The figure is generated using MATLAB R2011a (www.mathworks.com/).

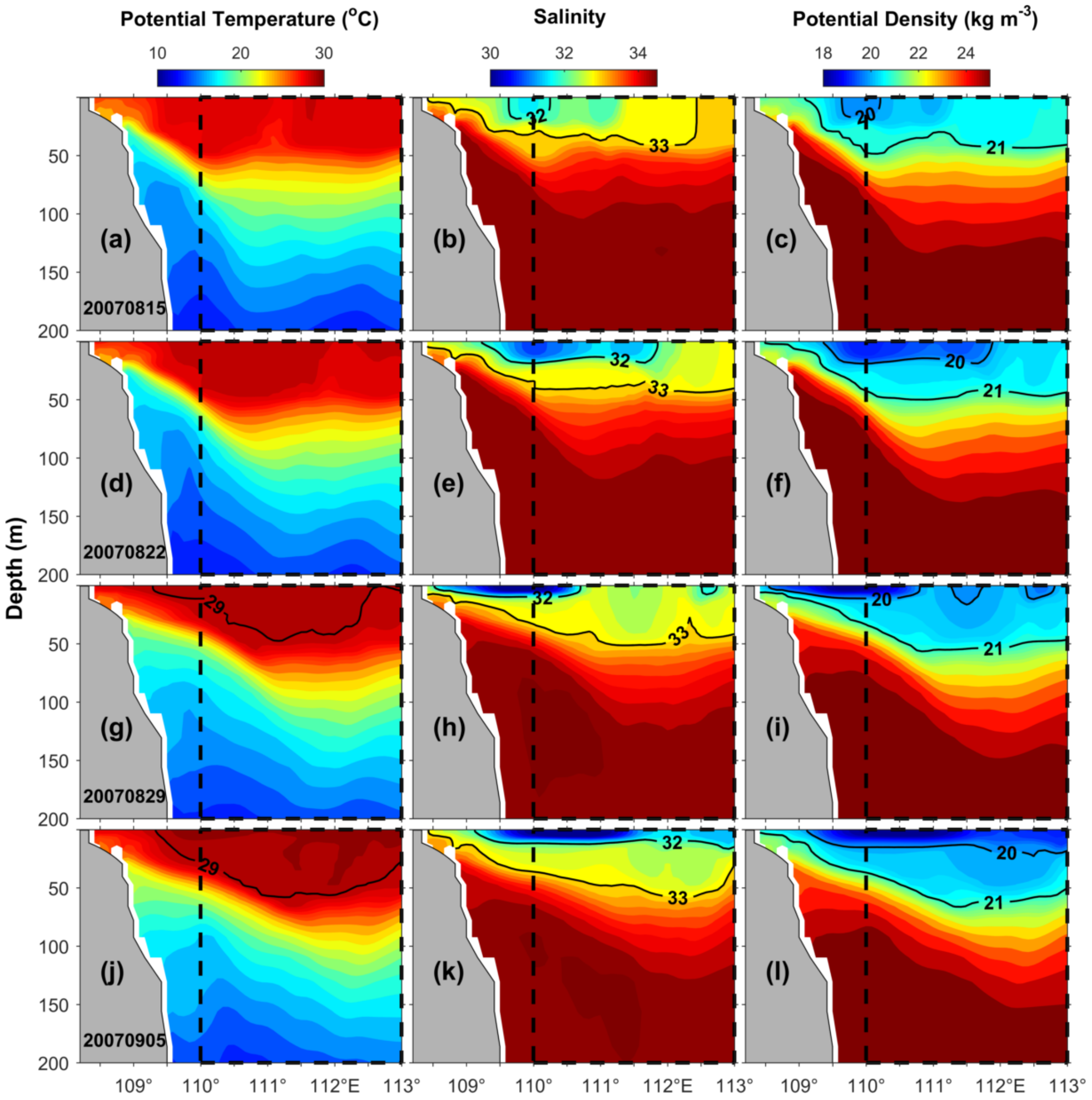

Figure S3. Weekly snapshots of sectional distribution of thermohaline properties along 11 °N based on CMEMS reanalysis. Similar format as Fig. 4. Each row is for a particular date which is shown in the leftmost panel. The figure is generated using MATLAB R2011a (www.mathworks.com/).

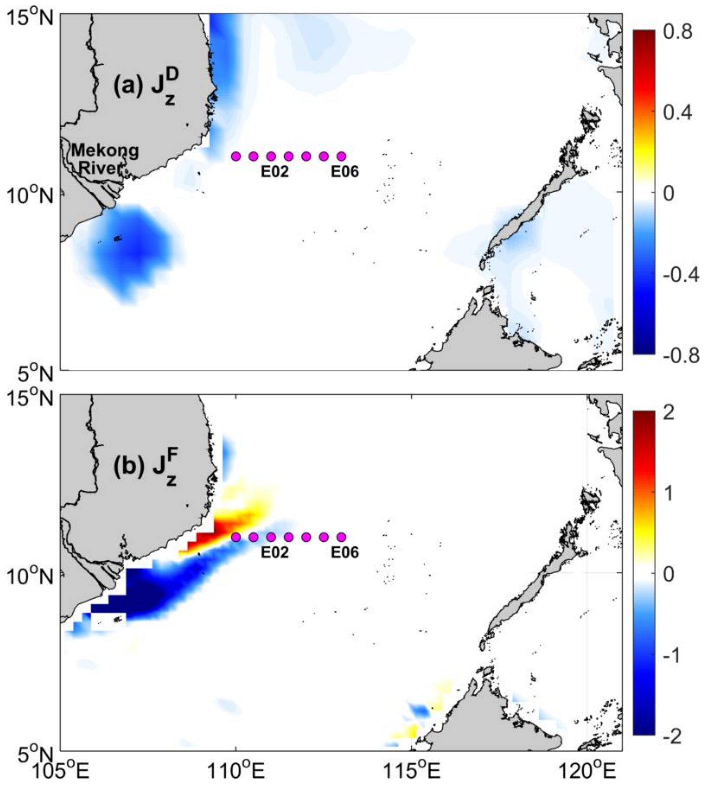

Figure S4. Vertical components of potential vorticity flux. PV flux associated with the (a) diabatic processes (J_z^D; in $\times 10^{-10}$ kg m^{-3} s^{-2}) and (b) frictional forces (J_z^F; in $\times 10^{-10}$ kg m^{-3} s^{-2}). Magenta dots denote stations along transect E marking the location of observed lens-shaped structure (E02–E06). The figure is generated using Matlab R2011a (www.mathworks.com/) with m_map package (www.eoas.ubc.ca/~rich/#M_Map).
